# Supplementary material for: Development of Urdu version of Chronic Respiratory Disease Questionnaire Self-Administered Standardized (CRQ-SAS); validity and reliability analysis in COPD patients
Source: PLoS One. 2023 Dec 28;18(12):e0293981. doi: 10.1371/journal.pone.0293981 (PMC10754428; doi:10.1371/journal.pone.0293981)
Supplement: Attachment — Submitted filename: Response to Reviewers.docx [file pone.0293981.s002.docx]

| **Comments from the Reviewer** | **Response from the Author** |
| --- | --- |
| Why did you change the author name and order after revision in the study? You mentioned that it is difficult to deal with the ethics committee for the title change. You should explain the reason for the change in author names without applying to the ethics committee. I think it would be appropriate to send your ethics committee approval document to editor. However, I think the title revision is necessary. Even in the conclusion part, you specified the result from the study “…we recommend health care professionals to use this tool to measure the disease-related quality of life in native COPD patients.”. It is sufficient for ethical compliance that the study method and participants are the same as in your ethics committee application. The one word change you make in the title does not create an ethical problem. I leave the final decision to the editor. My other review recommendations are listed below. If you make the revisions, I think your article is suitable for publication. The main point that needs to be clarified is that the author name and names order change. It must be proven that this has been done with ethics committee approval. | The Ethics Approval Document is already shared with the Editor of PLOS One in the very first attempt to submit the article.  This article is derived from Thesis work of a student named Momina Kashif (1^st^ Author in the article), with me (Danish Hassan, 2^nd^ Author) being her supervisor. The Ethical Review Committee Letter is issued by the name of Momina Kashif and the committee does not mention any other name on it. Hence the Ethical Committee does not regulate the names and their sequence that are meant to be written on manuscript produced form the thesis work. The Ethical Committee Letter is attached at the end for your instance also.  In reply to your second question about why the authors name was changed at this time is that here are 7 people that had intellectually contributed to finalization of this manuscript. I entertained only the first 5 in this manuscript based on the volume of their contribution. The author Hira Humayun has disagreed to financially contribute to this manuscript and asked for the removal of her name from it and hence I added a new name in replacement of her.  I have also changed the Title of Manuscript as per your recommendation. |
| Capitalize the first letters in the survey names on line 70. | The correction is done in line 70 |
| You should remove the phrase "progression" from the sentence in lines 316-320. You did not evaluate responsiveness. | The word is now removed |
| You attributed the low values in the factor analysis and reliability analysis results for items 3, 4 and 5 to socioeconomic level and lifestyle. “Walking (item 3), Performing chores, such as housework, shopping or grocery shopping (item 4), Participating in social activities, such as meeting with family, friends (item 5)”. You should explain the relationship of these items to the inference you mentioned. It is not clearly understood how walking is associated with socioeconomic level and lifestyle. Also, discuss this statement and add it to the discussion. | The patients that participated in this study were heterogenous in terms of the daily activities that they performed, occupation and life roles. Social activities vary across the socioeconomic status and walking is also linked to the nature of occupation might be the possible explanation for low item to total correlation of these three items only.  Socioeconomic status is the main factor that decides what sort of roles a person will have in this part of the world. For instance, managers of the company will have different walking patterns as compared to lower staff who has walk as a part of their job duty. The same is applicable to the other two factors (performing chores, such as housework, shopping, or grocery shopping & participating in social activities. |
